# Unraveling the mechanistic insights of sophorolipid-capped gold nanoparticle-induced cell death in *Vibrio cholerae*

**DOI:** 10.1128/spectrum.00175-23

**Published:** 2023-10-09

**Authors:** Sristy Shikha, Vineet Kumar, Ankita Jain, Dipak Dutta, Mani Shankar Bhattacharyya

**Affiliations:** 1 Biochemical Engineering Research and Process Development Centre (BERPDC), CSIR-Institute of Microbial Technology (IMTECH), Chandigarh, India; 2 Molecular Microbiology Laboratory, CSIR-Institute of Microbial Technology (IMTECH), Chandigarh, India; University of Minnesota Twin Cities, St. Paul, Minnesota, USA

**Keywords:** sophorolipid-capped gold nanoparticles, *Vibrio cholerae*, ROS, oxidative stress, apoptosis, membrane damage, DNA fragmentation

## Abstract

Cholera is one of the leading causes of mortality and morbidity worldwide. The treatment of cholera includes rehydration therapy, often combined with the administration of antibiotics (in severe cases). With the emergence and rapid spread of antimicrobial resistance, such microbial pathogens have become a compelling medical problem and a global threat. To combat the resistance to antibiotics, there is an urgent need for the discovery of novel and potent antimicrobial agents. The discovery of antimicrobial nanoparticles as an alternative to the existing antimicrobial regime has increased the hope for its use as one of the most promising tools against an array of microbial pathogens, including multidrug-resistant bacteria. Our recent report demonstrated that sophorolipid-capped gold nanoparticles (AuNPs-SL) exhibit potent antibacterial activity against *Vibrio cholerae*; however, the killing mechanism remained obscure. Metal nanoparticle-mediated killing of microbes occurs through non-specific mechanisms. To develop antimicrobial nanoparticles with better efficacy, knowledge of their specific mechanism of action is essential. In this work, we deduced the mechanistic insights of AuNPs-SL mediated cell death of *V. cholerae*. We observed that AuNPs-SL treatment evokes reactive oxygen species (ROS) formation. The surge in the ROS level triggers the overexpression of ROS-responsive genes, depolarization of the membrane, change in membrane permeability and leakage of intracellular proteins and DNA, depletion of ATP levels in the cell, DNA damage, and subsequent cell death. This study shows the possibility of use of AuNPs-SL as an alternative potential antimicrobial agent.

**IMPORTANCE:**

*Vibrio cholerae*, a Gram-negative bacterium, is the causative agent of a fatal disease, “cholera.” Prevention of cholera outbreak is possible by eliminating the bacteria from the environment. However, antimicrobial resistance developed in microorganisms has posed a threat and challenges to its treatment. Application of nanoparticles is a useful and effective option for the elimination of such microorganisms. Metal-based nanopaticles exhibit microbial toxicity through non-specific mechanisms. To prevent resistance development and increase antibacterial efficiency, rational designing of nanoparticles is required. Thus, knowledge on the exact mechanism of action of nanoparticles is highly essential. In this study, we explore the possible mechanisms of antibacterial activity of AuNPs-SL against *V. cholerae*. We show that the interaction of AuNPs-SL with *V. cholerae* enhances ROS production and membrane depolarization, change in permeability, and leakage of intracellular content. This action leads to the depletion of cellular ATP level, DNA damage, and subsequent cell death.

## INTRODUCTION

The causative agent of cholera, *Vibrio cholerae*, is a genetically versatile, Gram-negative, rod-shaped bacterium (1). *V. cholerae* can cross the acidic barrier of the stomach and colonize the small intestine, where it multiplies and secretes choleragen toxin (2) to cause cholera. Cholera is the second leading cause of mortality in children under 5 years of age and morbidity in adults (3). Out of the 200 serotypes of *V. cholerae* described, so far, only two, O1 and O139, are known to be the most virulent.

The treatment of cholera primarily includes rehydration therapy, often combined with the administration of antibiotics such as tetracycline, fluoroquinolones, and azithromycin for severe cases (4). In fact, oral rehydration was first introduced in the 1960s as a treatment against fluid loss and its replenishment that occur during acute diarrhea. It contains an iso-osmolar amount of glucose, electrolyte solution with base, and citrate, which is used to treat body water loss and metabolic acidosis. In decades following the first introduction of oral rehydration therapy, its preparation and administration have increased our capacity to control the outbreaks of diarrhea especially in developing countries and have largely remained effective.

In case of severe acute diarrhea, oral dose of antibiotic is administered as soon as the patient can tolerate oral medication. In most countries, doxycycline is recommended as the first line of treatment; however, azithromycin and ciprofloxacin are also given on a case-to-case basis. However, in the last few decades, extensive prescription and misuse of antibiotics enhanced the prevalence of antimicrobial resistance (AMR) cases, which poses a great threat to global health. In a recent study, it has been observed that *V. cholerae* may harbor as many as 40 different AMR encoding genes, making them capable of gaining resistance against as many as 22 antibiotics of nine different classes (5). Therefore, there is an urgent need to change the existing therapeutic strategies and to discover new antibiotics to combat AMR cases caused by *V. cholerae*.

Research outcomes in the past two decades have established nanoparticles as an alternative drug delivery as well as an antimicrobial agent to combat antibiotic-resistant microorganisms. Recently, metallic nanoparticles have been reported to exhibit antimicrobial activity against AMR bacterial strains (6). For example, nickel and Ni(OH)_2_ nanoparticles possess antimicrobial activities against multidrug-resistant (MDR) *Klebsiella pneumoniae* and *Escherichia coli* (7), copper nanoparticles against *Micrococcus luteus*, *Staphylococcus aureus*, *E. coli*, *K. pneumoniae*, *Pseudomonas aeruginosa*, and few fungal strains (8). The antimicrobial properties of silver nanoparticles against bacteria, fungi, and viruses are also well reported in the literature (9). Zinc oxide nanoparticles and gold nanoparticles (AuNPs) synthesized chemically are known to exhibit antibacterial activity against *V. cholerae* and are found to be efficient in lowering the bacterial burden (10). Zn is involved in immunocompetence and resistance of mucosa against infection and is a structural component of more than 200 enzymes. Silver nanoparticles are also potent against *V. cholerae* infection. Beside that, gold nanomaterials are considered to be one of the most suitable nanomaterials for biomedical applications, owing to their inherent biological inertness, well-established surface modification procedures, and facile and rapid preparation (11).

Biosurfactants produced by microbes also possess antimicrobial and antiadhesive activity and are amphiphilic in nature (12 – 14). Sophorolipid (SL) is a glycolipid biosurfactant with potential antifungal, antibiofilm, and hyphal growth inhibition activity (15). It shows antibacterial activity against Gram-positive bacteria but does not exhibit potent activity against Gram-negative bacteria (16). It is biodegradable and eco-friendly and therefore can be utilized in the greener synthesis of gold nanoparticles. We have demonstrated that sophorolipid-capped gold nanoparticles (AuNPs-SL) exhibit potent antimicrobial activity against both Gram-negative and Gram-positive bacteria but with higher efficacy against Gram-negative bacteria (17). However, AuNPs (uncapped) and SL independently lack antimicrobial activity toward Gram-negative bacteria. Although it is known that AuNPs-SL exhibit antibacterial properties, its molecular mechanism is obscure.

In this study, we report the plausible molecular mechanism of AuNPs-SL mediated killing of *V. cholerae*. We observe that AuNPs-SL treatment evokes reactive oxygen species (ROS) production, causes DNA damage, alters membrane potential, and depletes ATP levels that ultimately cause apoptotic cell death in *V. cholerae*.

## RESULTS

### AuNPs-SL evokes ROS production in *V. cholerae*

**Fig 1:**
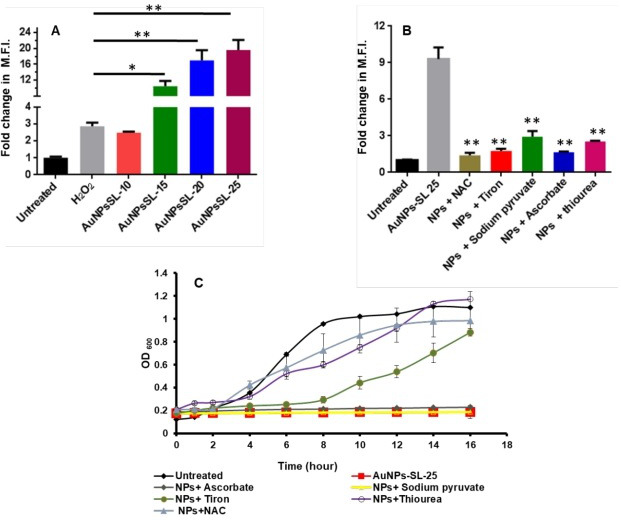
AuNPs-SL nanoparticles (NPs) evoke ROS production in *V. cholerae*. Fold change in mean fluorescence intensity (MFI) of H_2_DCFDA acquired by flow cytometer at different concentrations of AuNPs-SL (10, 15, 20, and 25 µg/mL) has been plotted (**A**). MFI of H_2_DCFDA acquired by flow cytometer at AuNPs-SL 25 µg/mL supplemented with ROS scavengers (NAC, tiron, sodium pyruvate, ascorbate, and thiourea) has been plotted (**B**). Growth curve of *V. cholerae* in the presence of AuNPs-SL-25 and different ROS scavengers (ascorbate, sodium pyruvate, tiron, thiourea, and NAC) (*n* = 3 ± standard deviation) has been plotted (**C**). Data are analyzed through paired *t*-test. *P =0.013 & **P < 0.001.

The nanoparticle-mediated killing of bacteria is known to be associated with ROS production and alteration in membrane structure and function that subsequently lead to cell death (18). To investigate whether AuNPs-SL treatment against *V. cholerae* causes ROS production, we measured ROS levels using a fluorescent dye, 2,7-dichlorodihydrofluorescein diacetate (H_2_DCFDA). ROS measurement was done in a dose-dependent manner of AuNPs-SL, from 10 to 25 µg/mL, based on minimum inhibitory concentration (MIC) calculations of AuNPs-SL against *V. cholerae* (MIC: 25 µg/mL) (17). Synthesis of AuNPs-SL was carried out according to the previously reported method (17). Fig. S1 shows the characterization of a representative batch of AuNPs-SL along with its transmission electron microscopy (TEM) image. With increasing concentrations of AuNPs-SL, it has been observed that the ROS levels also increase from 2.5-fold to 20.0-fold (Fig. 1A). To further validate the AuNPs-SL mediated ROS production, we used different ROS scavengers like N-acetyl-L-cysteine (NAC), tiron (Tr), ascorbate, thiourea (TU), and sodium pyruvate (SP) (19). Surprisingly, it has been found that NAC is highly efficient in scavenging AuNPs-SL mediated ROS levels, which is followed by Tr, ascorbate (all ~5-fold to 6-fold), TU, and SP (both ~3-fold) (Fig. 1B). Tr, SP, and TU scavenge ^-^O_2_, H_2_O_2_, and ^•^OH ROS species respectively. NAC replenishes the intracellular glutathione level or thiol-disulfide exchange to combat ROS-mediated redox imbalance of the cell (20, 21). Nevertheless, the supplementation of these scavengers in the growth medium rescues the growth of *V. cholerae* treated with AuNPs-SL (Fig. 1C). Although ascorbate and SP exhibit a significant ROS scavenging effect, neither of the compounds were able to rescue the growth of the dying cells (Fig. 1B and C). Similar phenomena were also observed with cells treated with silver nanoparticles (22). The mitigation of ROS levels and growth restoration in the presence of ROS scavengers clearly indicates that AuNPs-SL treatment evokes ROS production in *V. cholerae*.

### Dithiothreitol (DTT) restores the growth defect of AuNPs-SL treated cells

The restoration of growth in the presence of NAC make us stipulate the possibility of redox imbalance in the cell. DTT is a powerful reducing agent that has been shown to protect cells from oxidative stress and restore the cell viability (23). We also used DTT as a supplement to the AuNPs-SL treated cells and measured the ROS level. As a result, DTT treatment has been shown to reduce ROS levels in AuNPs-SL treated cells (Fig. S2A). To check whether DTT also restores growth, we performed spot assay in the presence of DTT. Likely DTT was found to restore the growth of AuNPs-SL treated cells (Fig. S2C). Being a strong reducing agent, we thought that DTT may alter the physical properties of nanoparticles and make them inactive. To rule out this possibility we checked the UV-spectrum of the nanoparticles after incubation with DTT. In DTT-incubated nanoparticles, no significant changes in UV spectra were found (Fig. S2D), that provides another line of evidence that AuNPs-SL evokes ROS in *V. cholerae*.

### AuNPs-SL alters the expression profile of ROS-responsive genes and the genes involved in stress responses

**Fig 2:**
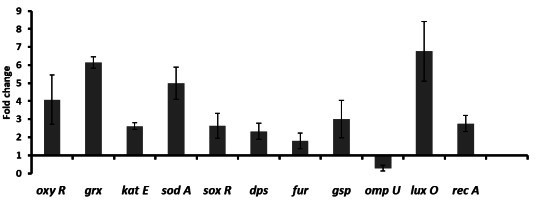
RT-qPCR in AuNPs-SL nanoparticle treatment. Fold change of selected genes in the presence of AuNPs-SL 25 µg/mL as compared to untreated samples. The data of biological duplicates have been plotted with ±standard deviation.

To mitigate the ROS, the living organisms possess their own line of defense and activate their ROS-responsive genes which act through different pathways. Therefore, the gene expression profile of selected genes has been checked in AuNPs-SL treatment by reverse transcription-quantitative PCR (RT-qPCR) (Fig. 2). A list of primer used for the study is provided in Table 1. The *soxSR* regulon is activated by ROS and regulates the expression of more than 40 genes. Superoxide radical upregulates the *soxR*, and the oxidized form of *soxR* acts as a transcription activator of *sodA* (24). *sodA* converts the superoxide anion (-O_2_) to O_2_ and H_2_O_2_ that ultimately gets reduced to H_2_O by catalase (25). The expression of *sodA* was upregulated ~5-fold in AuNPs-SL treated cells (Fig. 2). *sodA* converts superoxide to H_2_O_2_, which activates the OxyR (a dual regulator activated in the presence of H_2_O_2_), and OxyR further activates the genes involved in redox balance and metal homeostasis (26). In a study, it has been found that the *V. cholerae ΔoxyR* mutant is highly sensitive to ROS and has a growth defect even in the presence of rich growth media (27). An increase in intracellular H_2_O_2_ level triggers the expression of catalase (gene regulated by OxyR) (26). The genes *katG*, *grx (*glutaredoxin) , *fur*, and *dps* (Codes for DNA-binding protein, Dps) are also regulated by OxyR (28) (29) (30). Around (~) 4-fold upregulation of *oxyR* and five to six fold upregulation of hydroxyl peroxidase detoxification gene (*katE*) was observed in AuNPs-SL treated *V. cholerae* (Fig. 2). We also checked the expression level of OxyR regulated genes like *grx*, *dps*, and *fur*. The gene *grx* acts as an antioxidant and plays an important role in iron-sulfur cluster formation (29) (30). We found a ~6-fold increase in the expression levels of *grx* (Fig. 2) in treated cells. The growth of *Δdps* strain is significantly hampered in oxidative stress as well as in starvation, acidic stress, and metal stress (31). Dps sequestrates iron, limits the Fenton reaction (in Fenton reaction, there is oxidation of Fe^2+^ to Fe^3+^ in the presence of H_2_O_2_ to form hydroxyl free radical) and mitigates ROS levels (32) (33). The non-specific DNA binding of Dps protects DNA from ROS-mediated damage and the physical association of DNA with the toxic combination of Fe^2+^ and H_2_O_2_. The global regulator of iron homeostasis *fur* also protects cells from ROS by depleting free iron levels needed for Fenton reaction (34) (35). AuNPs-SL treatment increases the expression level of iron storage and *dps* and ferric uptake regulator (*fur*) by ~2.5-fold and ~2-fold, respectively (Fig. 2). ROS is known to cause damage to cellular DNA. DNA damage triggers the SOS response, and RecA upregulation is the hallmark of this process. We observed two fold upregulation of *recA* in the treated cells (Fig. 2). Beside that, in bacteria, the outer membrane acts as a selective barrier and limits the import of various toxic substances into the cell (36). For example, the *OmpU* is a major porin, which is highly conserved among *Vibrio* species. It facilitates movement of uncharged small molecules from the outer membrane to periplasmic space as well as involved in the adhesion/colonization of the bacteria (37). It has also been reported that in iron depletion, the expression of *omp* is downregulated as they act as a receptor of the siderophore complex and heme-compound transporter in *V. cholerae* (38). To understand the role of porins in AuNPs-SL treatment, we checked the expression of *ompU* and found its downregulation (Fig. 2). Downregulation of *ompU* facilitates the selective entry of nanoparticles and may also control the free iron levels to slow down the Fenton reaction. Moreover, the downregulation of *ompU* is also an indicator of impaired cell evasion and biofilm formation (37). Biofilm formation requires cell-to-cell communication and quorum sensing. The *luxO* gene regulates the cell-to-cell communication and cell signaling (required in different physiological processes like motility, colony formation, and biofilm formation) (39). *luxO* is upregulated by seven fold (Fig. 2). Its upregulation depicts the possibility of lifestyle switching *in V. cholerae* upon AuNPs-SL treatment. For survival in stressful niches, *V. cholerae* employs remodeling of outer membrane assembly and activates secretion systems (which is required for the export of several proteins like chitin, cholera toxin, and protease) (40) (41), and activation of the general secretion pathway (gsp) genes is required for this. AuNPs-SL treatment upregulates the expression of *gsp* by three fold (Fig. 2).

**TABLE 1:** List of primers used in the RT-qPCR^*a*^

Primers		Sequence 5′−3′	Tm (°C)	Amplified product size (bp)
*dps*	F	TGCACAGCTTTAGCGGTTAC	55.96	119
	R	TTGTTGGTGGAGCAGAATGC	56.11	
*oxyR*	F	GCACTTATTGTTGCGCGAAG	55.95	123
	R	CCCACCACATAGCTCTCCAT	55.86	
*grx*	F	TACCGCTTATGCAACGCAAA	55.95	110
	R	TTCCAGCCGATCATCAAACG	55.74	
*gsp*	F	GCTCTCTCAAGCGGAGTTTG	56.06	121
	R	TTCCGCCAGTGAGAAGAAGT	55.98	
*luxO*	F	ACTGCGGGTCACAGTGAATA	56.06	135
	R	GAGTCAATGGTGCGGTACAC	56.05	
*katE,*	F	GGTGATTAAGGCCGCACAAA	56.19	112
	R	AGCCATTTGCTGCCAATCTT	55.74	
*ompU*	F	CGGTGACAAAGCAGGTTCAA	56.07	120
	R	CGTACGCGAGAGTTGTCTTG	56.23	
*recA*	F	GGGCGTGAATATCGATGAGC	56.07	102
	R	ATGACATCCACAGCACCAGA	56.02	
*sodA*	F	GTGAACACCTTTGGCTCTGG	56.13	106
	R	CGGCAACCACATCCATCAAT	55.96	
*soxR*	F	GAAGGTCTCAGCGTTGCATT	55.94	140
	R	AGGTCAGTCCGACCATTTGT	55.95	
*fur*	F	ACAGCCAGAGTGCCAACATA	56.32	130
	R	ACGAGTCACGATACCAGCAT	55.96	
*luxR*	F	GATCCAAACCGCTCAGCATT	55.98	139
	R	TGGCGTTACGCAAGTGATTT	55.82	

^*a*^ F: forward primer, R: reverse primer, Tm: melting temperature

In conclusion, the upregulation of ROS-responsive genes *sodA*, o*xyR*, *katE*, etc, iron storage protein *dps*, *fur*, SOS response gene *recA*, outer membrane gene *ompU*, and genes involved in quorum sensing (*luxO*) and secretion systems *gsp* indicates that AuNPs-SL treatment evokes ROS production, causes DNA damage, and alters cell-to-cell communication to counter its toxic effects.

### AuNPs-SL treatment causes DNA damage and apoptosis in *V. cholerae*

**Fig 3:**
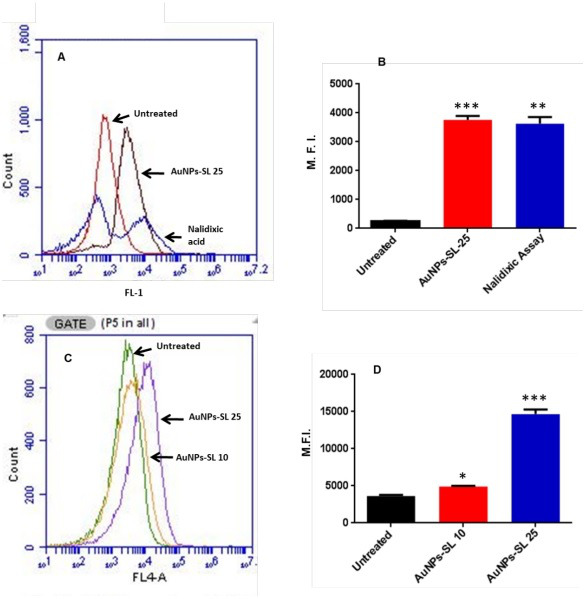
TUNEL and apoptosis assay. Graphical representation of fluorescein-labeled dUTP cells at AuNPs-SL-25 (**A**). Mean fluorescence intensity (MFI) of fluorescein-labeled dUTP acquired by flow cytometer at AuNPs-SL-25 has been plotted (**B**). Nalidixic acid is taken as a positive control (*n* = 3 ± standard deviation (SD)) *P < 0.01. Data analysis was performed using paired *t*-test. Measurement of apoptosis upon AuNPs-SL treatment using annexin affinity assay. Graphical representation and MFI of annexin V allophycocyanin conjugate acquired by flow cytometer at AuNPs-SL 10 and 25 µg/mL have been plotted (C and D). MFI of annexin positive cells (*n* = 3 ± SD). Data analysis is done through paired *t*-test. *P < 0.01, **P = 0.013 for AuNPs-SL10 µg/mL, ***P < 0.001 for AuNPs-SL-25 µg/mL.

ROS measurement and RT-qPCR data clearly demonstrate that AuNPs-SL treatment of *V. cholerae* evokes ROS generation. Induction of ROS damages DNA by creating lesions in bases, sugar, and DNA protein cross-links within the single- and double-strand bases of DNA (42). Overexpression of *dps* and *recA* (Fig. 2) (the DNA protecting and SOS response genes, respectively) further confirms the possibility of DNA damage in AuNPs-SL treated cells. To probe this, we performed terminal deoxynucleotidyl transferase-mediated dUTP-X nick end labeling (TUNEL) assay. In this assay, the fluorescence is measured from the free 3′-OH of the damaged DNA (synthesized from fluorescein-labeled dUTP by exogenously supplied terminal deoxynucleotidyl transferase). Thus, when cellular DNA is fragmented, the incorporation of labeled dUTP increases the fluorescence inside the cells. The AuNPs-SL treatment (at 25 µg/mL) increases the fluorescence intensity by 20-fold (Fig. 3A and B), which manifests severe DNA damage and fragmentation. Further, DNA fragmentation initiates the programmed cell death or apoptosis. Therefore, to probe the AuNPs-SL mediated apoptotic cell death, we performed apoptosis assay by using annexin V allophycocyanin conjugate. Annexin V has a higher affinity for phosphatidylserine, which becomes exposed to the outer leaflet in the cell membrane undergoing apoptosis. We observed a ~5-fold increase in the fluorescence intensity of annexin V in treated cells (25 µg/mL AuNPs-SL); however, the effect of a lower amount of AuNPs-SL (10 µg/mL) was not significant (Fig. 3C and D). Nevertheless, TUNEL assay and apoptosis assay exhibit apoptotic cell death of *V. cholerae* upon AuNPs-SL treatment.

### Iron supplementation rescues AuNPs-SL mediated growth defect in *V. cholerae*

**Fig 4:**
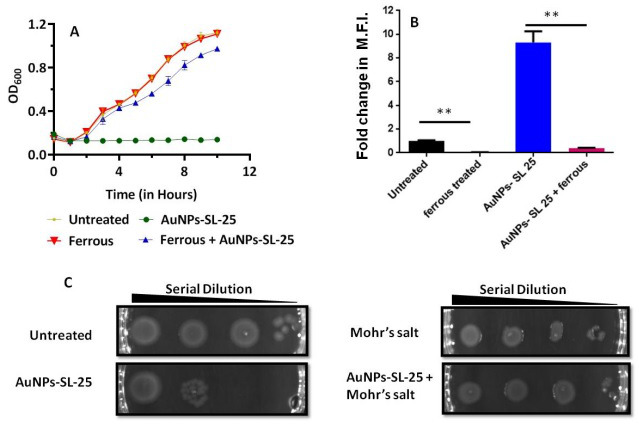
Iron supplementation rescues the growth and decreases ROS levels. The growth curve in the presence and absence of AuNPs-SL-25 µg/mL and iron supplement (Mohr’s salt) (**A**). Fold change in MFI of H_2_DCFDA shows that iron decreases ROS levels (**B**). Spot assay in the presence of AuNPs-SL-25 µg/mL and Mohr’s salt. **P value < 0.01 calculated through paired *t*-test (**C**).

In bacterial cells, iron uptake and storage are critically controlled and regulated by cellular physiology and homeostatic mechanism. To mitigate the elevated ROS levels caused by AuNPs-SL, *dps* (an iron storage protein) and *fur* (iron uptake regulator) genes have been upregulated (Fig. 2). They counter the free iron levels to inhibit free radical formation by Fenton reaction. This leads to the imbalance in the availability of iron for other physiological processes. Iron is an essential micronutrient for the growth and metabolism of microorganisms and works as a cofactor for various enzymes, viz, [Fe–S] cluster containing ferredoxins, heme-containing cytochromes, and fumarases (43). Therefore, the effect of external iron supplementation was checked in *V. cholerae*. Growth studies revealed that iron supplementation rescues the growth of AuNPs-SL treated cells (Fig. 4A). In addition to that, iron supplementation significantly decreased ROS production in the treated cells (Fig. 4B). Since the Fe-S clusters are the integral part of the electron transport chain (ETC) and their malfunctioning causes ROS generation (44), the supplementation of iron may repair the impaired Fe-S cluster assemblies in AuNPs-SL treatment that further decreases the ROS formation and the cells regained their growth and viability. Since increased ROS levels cause the overexpression of genes involved in iron-sulphur cluster formation, iron sequestration, and storage and iron homeostasis and protect the cell from ROS toxicity by depleting free iron required for Fenton reaction, we checked the expression of few selected genes, e.g., *sod A*, *rec A*, *lux O*, and *oxy R* in AuNPs-SL treated *V. cholerae* cells in the presence of Mohr’s salt (ammonium iron(II) sulfate). The expression level of all the selected four genes was downregulated in Mohr’s salt supplemented in comparison to the AuNPs-SL treated cells (Fig. S3). Mohr’s salt, which is resistant to environmental oxidation, is more capable of Fe^2+^ stabilization, prevention of Fenton reaction, and reduction of ROS stress and is capable of rescuing the cells from ROS toxicity.

### AuNPs-SL treatment damages the cell membrane and causes membrane leakage in *V. cholerae*

**Fig 5:**
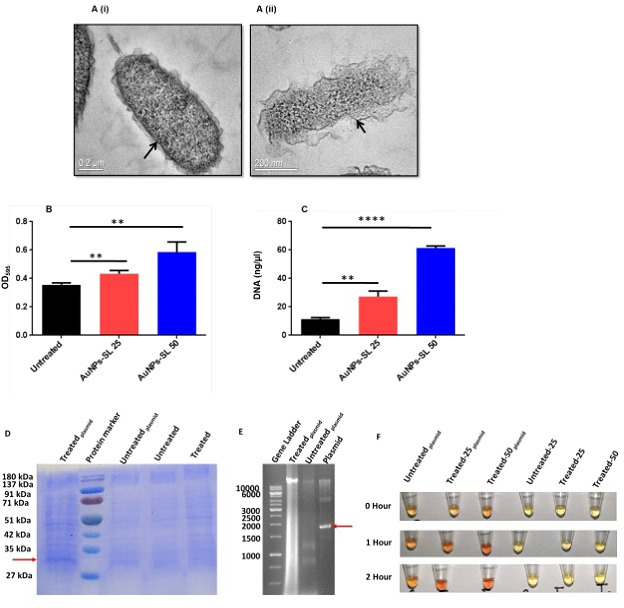
AuNPs-SL cause membrane damage and leakage of intracellular contents. TEM images of *V. cholerae* (**A**) in the absence (**A**i) and presence (**A**ii) of AuNPs-SL. Absorbance at 595 showing the leakage of protein (**B**). Nanodrop readings of DNA concentration at varying concentrations of AuNPs-SL (25 and 50 µg/mL) (**C**) (*n* = 3 ± SD). The data were analyzed using an unpaired *t*-test **P< 0.01 and ****P < 0.0001. SDS-PAGE gel image of total protein isolated from untreated and treated cells without and with β-lactamase plasmid (**D**). Agarose gel electrophoresis of untreated and treated cells containing β-lactamase plasmid (**E**). β-lactamase activity of the above-mentioned cells and conditioning at different time intervals (**F**).

The bactericidal activity of AuNPs-SL against *V. cholerae* suggests the interaction of the AuNPs-SL with the cell membrane. Therefore, we checked the architecture of the outer surface and cell membrane of *V. cholerae* by TEM. The TEM images showed the extensive damage to outer surface and ruptured membrane in the AuNPs-SL treated cells as compared to the untreated cells (Fig. 5Ai and Aii). When the cell membrane is damaged, there is a possibility of leakage of intracellular components such as proteins and DNA in the surrounding environment. Therefore, we measured the amount of protein and DNA in the extracellular media. Likely, in AuNPs-SL treated cells, we observed a concentration dependent increase in the release of protein and DNA content (Fig. 5B and C). Treatment with 25 and 50 µg/mL of AuNPs-SL resulted in two fold and four fold increase in the DNA leakage, respectively. To strengthen our findings, we also measured the protein content using β-lactamase plasmid in *V. cholerae*. The molecular weight of β-lactamase is approximately between 30 and 40 kDa (45). In the SDS -PAGE gel image, a distinct and obvious protein band is present below 35 kDa from total protein isolated from the treated_plasmid_ cells which are very faint in untreated_plasmid_ cells and completely missing in untreated and treated cells without plasmid. Agarose gel images (Fig. 5E) of equally loaded DNA (by volume 20 μL) clearly depict the higher DNA amount with smear in AuNPs-SL treated_plasmid_ cells as compared to unreated_plasmid_ cells. The smear throughout the lane of treated_plasmid_ also indicates the DNA fragmentation. To further validate our findings, we performed nitrocefin-based chromogenic assay. Nitrocefin, a chromogenic cephalosporin, becomes red when β-lactamase hydrolyzes its amide link. It is frequently used for the detection of microbes producing beta-lactamase enzymes. Treated_plasmid_ cells have a higher intensity of red color than untreated plasmid cells; however, no color change occurs in cells lacking plasmid (Fig. 5F). The intensity of red color for samples increases with increasing time. These experiments clearly demonstrate that AuNPs-SL treatment of *V. cholerae* leaks the intracellular contents. The membrane damage also causes osmotic shock to the cells (30) (46). To protect the cells from osmotic shock, the sodium and potassium ions play a very important role. Therefore, we measured the concentration of these ions using inductively coupled plasma mass spectrometry (ICP-MS) and observed that AuNPs-SL treatment led to an increase in K^+^ and Na^+^ (Table 2). TEM imaging, extracellular protein and DNA content measurement, and the increased level of K^+^ and Na^+^ show that AuNPs-SL severely damages the cells to exhibit apoptotic cell death.

**TABLE 2:** Ion concentration measurement under AuNPs-SL stress

Ion concentration	Control	AuNPs-SL-10
Iron	146 ± 15	299 ± 19
Sodium	2058 ± 54	4076 ± 33
Potassium	2655 ± 31	4434 ± 36
Calcium	1591 ± 18	1583 ± 17

### AuNPs-SL causes membrane depolarization in *V. cholerae*

**Fig 6:**
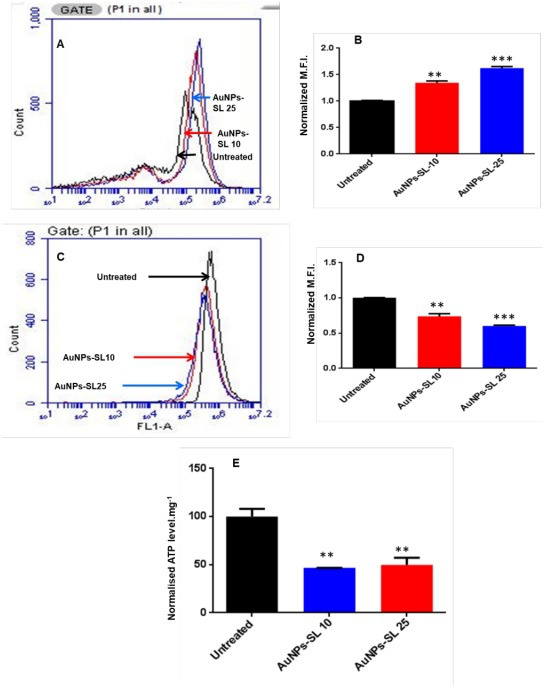
AuNPs-SL depolarizes the membrane. Concentration dependent increase in normalized MFI of DiBAC4 indicates plasma membrane depolarization (A and B). A concentration dependent decrease in MFI of DiOC2 indicates plasma membrane depolarization (*n* = 3 ± SD) (C and D). The data was analyzed using paired *t*-test. **P < 0.01 and ***P < 0.001. Relative ATP measurement at varying concentrations (10 and 25 µg/mL) of AuNPs-SL nanoparticles (**E**). The normalized relative light units) are plotted (*n* = 3 ± SD).

Severe membrane damage and intracellular leakage in AuNPs-SL treatment may alter the other physical features of the membrane. It is evident that both ROS production and membrane damage cause membrane depolarization (47). To investigate whether AuNPs-SL also depolarizes the membrane in *V. cholerae*, we measured the membrane potential in the varying concentrations of AuNPs-SL using two different dyes, DiBAC_4_ and DiOC_2_. DiBAC_4_ is an anionic lipophilic bis-oxonol dye. When the membrane depolarizes, as the membrane potential shifts from negative to positive, the concentration of the dye entering the cells increases (i.e., the higher the membrane depolarization, the higher the oxonol fluorescence intensity). In our experimental setup, when the *V. cholerae* cells were treated with 10 and 25 µg/mL of AuNPs-SL, membrane depolarization increased by 13% and 16%, respectively (Fig. 6A and B). Also with DiOC_2_, there is a concentration-dependent change in the membrane potential as indicated by the intensity of DiOC_2_ (Fig. 6C and D); the change in fluorescence intensity of DiOC_2_ is directly proportional to the loss of membrane potential of the cells (48). ROS formation is linked with membrane depolarization (47). To demonstrate this link, we used DTT supplementation because DTT rescues the cells from ROS and also helps in decreasing membrane depolarization (49). Surprisingly, it has been observed that DTT rescues the AuNPs-SL treated cells, but it is not able to restore the membrane depolarization (Fig. S2B).

### AuNPs-SL decreases ATP production in *V. cholerae*

ETC components possess Fe-S clusters and are located in the membrane (50). ROS formation, growth rescue in iron supplementation, depolarized membrane, and altered membrane architecture manifest the impairment of electron transport chain. The aforementioned impairment of ETC in AuNPs-SL treated *V. cholerae* prompted us to investigate whether AuNPs-SL treatment causes energy crisis in the cell or not. We measured the intracellular ATP level using a luciferase based ATP bioluminescence assay and observed a ~50% reduction in ATP levels in AuNPs-SL treated cells. However, no dose-dependent decrease in ATP levels was observed in the treatment range (10 and 25 µg/mL) of AuNPs-SL (Fig. 6E).

## DISCUSSION

**Fig 7:**
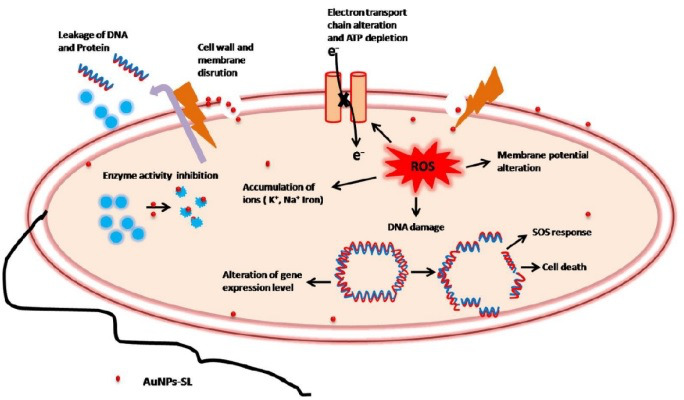
Schematic representation of mechanistic insight of AuNPs-SL mediated killing of *V. cholerae.* AuNPs-SL interacts with the membrane to cause membrane damage and accumulates in the cell. Accumulation of nanoparticles causes ROS formation which has various physiological consequences like oxidative damage to DNA (which further lead to DNA fragmentation and apoptosis), activation of ROS-responsive genes, and impaired iron homeostasis. Membrane damage and ROS together lead to osmotic imbalances, leakage of intracellular content (protein and DNA), alteration in proton motive force, and membrane potential which ultimately depletes intracellular ATP content.

The rise in cholera epidemics has become a major concern due to the emergence of multidrug-resistant bacterial strains and the limited availability of novel pharmaceutical interventions in the market (51). Hence, it is imperative to employ novel approaches in order to discover and cultivate the next cohort of pharmaceuticals aiming to manage cholera infections. AuNPs are Food and Drug Administration-approved metallic nanoparticles with promising medical applications (52) (53). AuNPs are used in drug delivery and photothermal, therapeutic, radiosensitizing, and gene transfection (54). In our previous work, we demonstrated the antibacterial, antibiofilm, and growth inhibitory effect on non-dividing cells of AuNPs-SL (17). AuNPs-SL were also effective against *V. cholerae* grown in virulent conditions, and IC_50_ was found to be at AuNPs-SL-50; 90% of inhibition was observed at AuNPs-SL-100 and complete eradication at AuNPs-SL-200 (Fig. S4A). The current work aims to uncover the mechanistic insights of AuNPs-SL mediated cell death of *V. cholerae*. Like antibiotic stress (55), we observed that AuNPs-SL treatment preliminarily causes ROS formation (Fig. 1). The concentration-dependent increase of ROS levels in the AuNPs-SL nanoparticle treatment (Fig. 1A), the growth restoration, and decrease in ROS levels in the presence of ROS scavengers (NAC, ascorbate, tiron, SP, and TU) (Fig. 1B and C) demonstrate that AuNPs-SL evokes ROS in *V. cholerae*. Induction of ROS leads to the cascade of events in the cells. Primarily, it induces ROS-responsive genes to counter the ROS levels, e.g., *oxyR*, *soxR*, *katE*, *sodA*, *fur*, and *dps* (56). In this study, we observed the upregulation of the selected genes (*sodA*, *oxyR*, *katE*, *dps*, *fur*, and *grx*) involved in the intracellular ROS mitigation (Fig. 2). Prior studies suggest that ROS production causes DNA damage, membrane damage, impaired Fe-S cluster biogenesis, lowered ATP production, etc. (57) (58) (59). RecA is the hallmark of SOS response (60). The upregulation of *recA* in treated cells probed the possibility of DNA damage and subsequent apoptotic cell death in the treated cells that has been further exhibited by TUNEL assay (Fig. 3A and B) and apoptosis assay (Fig. 3C and D). Rescue of AuNPs-SL treated *V. cholerae* by iron supplementation shows the possibility of impaired Fe-S biogenesis, which further slows down the ETC and subsequent leakage of electrons (Fig. 4). The leakage of electrons from ETC could be the major reason for ROS generation. Impairment of ETC also manifests energy crisis in the cell (61). ROS causes various physiological assaults to the cell, Therefore, we were curious to know the process of entry of AuNPs-SL in to the cells and their effects. The bacterial membrane is selective in nature; thus, downregulation of porin *ompU* indicates the resistance of the AuNPs-SL into the cells. To understand the nanoparticle and membrane interaction, the TEM imaging was done. TEM images showed that AuNPs-SL cause physical damage to the membrane (Fig. 5Aii) by interacting with the outer membrane of *V. cholerae*. Physical damage of membrane led to uncontrolled entry of nanoparticles inside the cell. Damage to the outer surface cause osmotic imbalances in the cell, which lead to the leakage of intracellular content like DNA and proteins (Fig. 5). The upregulation of *luxO* and *gsp* (Fig. 2) further depicts that the nanoparticles manipulate the cell-to-cell communication and signaling to combat stress by switching the lifestyle of bacteria. The relation between nanoparticle treatment and iron homeostasis perturbation is a matter of future investigation to gain insights into the metabolic rewiring of *V. cholerae* due to AuNPs-SL treatment. The molecular mechanism of AuNPs-SL mediated killing of *V. cholerae* is summarized in the model (Fig. 7). The cell death model presented in Fig. 7 depicts the interaction of the AuNPs-SL with the cell surface, membrane damage, their entry in to the cell, and subsequent cascade of events. Membrane structure alteration and damage has various consequences like the entry of more AuNPs-SL, osmotic imbalance, and intracellular content leakage. The increased titer of nanoparticles affects various physiological processes, e.g., ROS generation by impairing Fe-S biogenesis. ROS induces the cascade of events such as DNA damage and apoptotic cell death. The damaged membrane induces the intracellular leakage of proteins and DNA and alters osmotic balance. Damaged membrane also affects ETC, causes membrane depolarization, and ultimately lowers the energy state of cells, which is the lifeline of every living organism (Fig. 6).

Few studies using metallic nanoparticles have employed silver, zinc, or selenium nanoparticles as antimicrobials against *V. cholerae* (62). Chatterjee et al. used gold nanoparticles of different shapes and sizes for the eradication of biofilm of *Vibrio cholerae* in an *in vivo* animal model. They found inhibition of biofilm and compromised production and structure of cholera toxin, and reduction of fluid accumulation in infected mice treated with nanoparticles (63). Similarly, we speculate that AuNPs-SL might inhibit the expression and production of several vital proteins having crucial roles in survival of *V. cholerae*.

Greener AuNPs-SL have the ability to reduce cell viability at all stages of the *V. cholerae* life cycle, including active (planktonic) and latent (biofilm and non-dividing) cells (previous findings) and virulent condition (this study). Therefore, AuNPs-SL can be used as an alternative therapy at any stage/form to combat *V. cholerae* infection. These nanoparticles interact with the cell membrane, create ROS surge, upregulate genes. deplete ATP, cause DNA damage, alter membrane potential, and initiate loss of cellular content, all of which influence *Vibrio’s* survival. However, the *in vivo* study is still remaining to establish it as a formulated drug.

This study provides a new, inexpensive alternate method to reduce the infectious dose of pathogen contaminated water and potential of an alternative *in vivo* therapy against cholera infection. Further studies are being carried out to find out the toxicity (both acute and long term) and carcinogenicity and clearance of the nanoparticle in the animal model. Besides that, the efficacy of the AuNPs-SL is being tested for lowering of toxin production and mutagenicity. Further efficacy of these nanoparticles could be tested against MDR strain of *V. cholera*e. Further, we are also interested to understand the cross-talk of iron homeostasis and metabolic rewiring of bacteria in nanoparticle treatment. This study will further strengthen its use as a combinatorial antibiotic therapy.

## MATERIALS AND METHODS

### Growth conditions and cell viability

Gram-negative *V. cholerae* EL Tor N16961 strain was used in this study. In all the experiments, unless mentioned otherwise, growth was done at 37°C in Luria broth (LB) at 200 rpm in an orbital incubator shaker. For experiments, the nanoparticle treatment was given at an optical density at 600 nm (OD_600_) of 0.2. To perform growth assays, Bioscreen C growth curve machine was used. In the growth assays, log phase cells were diluted 1,000 times, and 100 µL of diluted culture was added to the honeycomb plate with the indicated supplements, and volume was made up with sterile LB to 200 µL. OD_600_ was measured at an interval of 1 hour, and a graph was plotted. For spot assay, an LB agar plate with different concentrations of AuNPs-SL (25 µg/mL), Mohr’s salt (1.5 mM), and a combination of the two was prepared. *V. cholerae* cells at log phase were diluted to a ratio of 1:10^2^, 1: 10^4^, and 1:10^6^, and 10 µL of each was spotted incubated at 37°C overnight. The images of plates were taken and representative images are shown.

### Nanoparticle synthesis

Synthesis of AuNPs-SL was done as mentioned earlier (17). Briefly, the synthesis of AuNPs-SL involves the addition of 40 µL of sophorolipid (100 mg/mL) in 10 mL of a chloroauric solution with a concentration of 400 µg/mL (pH 5.5 ± 0.2) with a few drops of freshly prepared sodium borohydride solution (NaBH_4_, 100 mM).

### Measurement of ROS

ROS was measured using ROS-sensitive fluorescent probe H_2_DCFDA (Sigma Aldrich). Once the dye enters the cell, it senses the ROS and gets oxidized to form the fluorescent product, 2′,7′-dichlorofluorescein (64). Concentration of AuNPs-SL was taken in the range of 10–25 µg/mL based on our previous finding of antimicrobial activity of AuNPs-SL against *V. cholerae*. Cells were grown until OD_600_ of 0.2 and were treated with different concentrations of AuNPs-SL. After treatment, the cells were spun down and washed thrice with phosphate buffered saline (PBS) and stained with H_2_DCFDA (10 µM) for 1 hour at 37°C. After incubation, the fluorescence of 20,000 cells was measured using a flow cytometer (Accuri C_6_) in the FL1 channel. The normalized mean fluorescence intensity (MFI) of three independent experiments was plotted with ±SD. The ROS was also measured by supplementing ROS quenchers. The cells were treated with AuNPs-SL-25 and supplemented with 10 mM of respective quenchers.

### RT-qPCR

Genes were selected on the basis of their role in intracellular ROS scavenging (established by published findings). To check the expression profile of the selected genes in the presence of AuNPs-SL, RT-qPCR was performed. At higher concentrations of AuNPs-SL, isolated RNA shears/degrades; therefore, a lower concentration of AuNPs-SL (10 µg/mL) was used for the study. RNA extraction was done using the Trizol reagent method (65), and cleanup was done using Qiagen Kit. The RNA was checked for its quality and quantified using Nano-Drop spectrophotometer. One-step SYBER Green Master Mix reaction mixture (Invitrogen) was used to perform the qPCR reaction in a Fast Real-time PCR system (Applied Biosystem) with 200 ng of RNA per reaction. The data of triplicate experiments of biological duplicates are plotted with SD.

### TUNEL assay

TUNEL assay was performed to check nanoparticle-mediated DNA fragmentation in *V. cholerae* using a kit. Briefly, cells were grown with AuNPs-SL (25 µg/mL) and nalidixic acid (5 µg/mL) for 3 hours. After treatment, the cells were harvested and washed with PBS (pH 7.2), fixed with 2% formaldehyde (15 minutes on ice), and treated post-fixation with 70% ethanol. The fixed cells were permeabilized with permeabilization buffer (0.1% Triton X-100 and 0.1% sodium citrate), while being kept on ice for 2 minutes. After washing, the cells were resuspended in 50 µL of the solution containing enzyme (terminal deoxynucleotidyl transferase) and reaction mixture in the ratio of 1:9. After 1 hour of incubation at 37°C, the cells were washed again and resuspended in PBS. The flow cytometer data of 20,000 cells were acquired using an FL1 laser of Accuri C_6_ flow cytometer and the mean fluorescence intensity of three independent experiments was plotted with SD.

### Apoptosis assay

To check the nanoparticle-induced apoptosis in *V. cholerae*, we performed an annexin affinity assay. In brief, the cells were treated with AuNPs-SL and were washed twice with PBS buffer. Staining was done with annexin V allophycocyanin conjugate (5 µg/mL) for 20 minutes. After staining, the cells were washed with PBS to remove the unbound stain and were suspended in PBS. The fluorescence intensity of 20,000 cells was measured using the FL4 filter of the Accuri C_6_ flow cytometer. The MFI values of three independent experiments were plotted with SD.

### Transmission electron microscopy

To check the alterations in the ultrastructure of *V. cholerae* membrane in AuNPs-SL treatment, TEM was performed by following protocol (66) with slight modifications. In brief, both treated (AuNPs-SL 25 µg/mL for 3 hours) and untreated cells were pelleted and washed with PBS twice. Fixation was done with modified Karnovsky’s fixative containing 2% (vol/vol) glutaraldehyde and 2% (vol/vol) paraformaldehyde in 0.1-M sodium cacodylate buffer (pH 7.2) at 4°C for 2 hours (67). Fixation was followed by washing with PBS and post-fixation treatment with 0.2-M sodium cacodylate buffer and osmium tetroxide [200 µL of 2% (wt/vol)] at 4°C for 90 minutes. The sample was then washed thrice with 0.1-M sodium cacodylate buffer and resuspended in the same buffer containing 2% agarose. A thin section was cut using a microtome followed by its gradual dehydration with acetone solution (once at 30%, 50%, 70%, and 90% and twice at 100%). Finally, the sections were examined under TEM in JEOL-2100. The representative images have been shown.

### Measurement of leaked cellular contents (protein and DNA)

To determine the leakage of cellular contents (protein and DNA), the cells were treated with different concentrations of nanoparticles (AuNPs-SL, 25 and 50 µg/mL) followed by a Bradford assay and nanodrop readings to quantify the protein and DNA contents of the samples, respectively. For measurement of protein, 1 mL of cell sample (treated and untreated) was withdrawn after 2-hour incubation of AuNPs-SL treatment. The cells were pelleted, and the supernatant was used for protein quantification by the Bradford method. For measurement of DNA, untreated and treated cells were harvested, and DNA was extracted from the supernatant as per manufacturer instruction (ZR Fungal/Bacterial DNA Kit catalog no. D6005). DNA quantification was done by Nanodrop 1,000 spectrophotometer (Nanodrop Technologies Inc., USA). For agarose gel analysis, DNA was isolated manually from the supernatant from untreated and treated (AuNPs-SL-25) by precipitating the supernatant with chilled isopropanol followed by washing with ethanol. Isolated DNA was quantified and electrophoresed on 1% agarose gel. Isolation of the total protein was carried out after the completion of the treatment time. In a typical procedure, samples were spun down, and the supernatant was collected for further study. An equal volume of solution, i.e., a mixture of methanol and chloroform (4:1), was added to the cell soup with proper mixing and was kept at 4°C for 5 minutes. It was then centrifuged at 7,000 rpm for 10 minutes at 4°C. The upper layer was carefully discarded without disturbing the ring/interface. An equal volume of methanol was added, vortexed gently, and centrifuged at 10,000 rpm for 10 minutes at 4°C. The pellet was washed again and dried to remove residue from the solvent. It was resuspended into Tris-NaCl buffer and quantified using Bradford assay. The samples were analyzed on a 12% SDS-PAGE gel.

β-Lactamase assay was carried out using nitrocefin reagent. Nitrocefin is a β-lactam that changes color when a β-lactamase breaks it down. Fifty microliters of untreated and treated cell supernatant was mixed with the 2-µL nitrocefin solution (5 mM) and incubated in the dark to observe the color change.

### Membrane depolarization assay

To perform membrane depolarization assay, the untreated and AuNPs-SL treated were grown for 3 hours. After incubation, the cells were centrifuged at 4,000 rpm for 10 minutes, and the cell pellet was washed with PBS. The washed cells were resuspended in 10 µM DiBAC_4_ (1-mM DMSO) and incubated for 30 minutes at 37°C in the dark. The cells were pelleted and washed with PBS to remove the extra stain. Finally, the cells were suspended in PBS and the fluorescence intensity of 20,000 cells was measured using an FL1 laser of Accuri C_6_ flow cytometer.

Loss of membrane potential was also analyzed by using dye 3,3′-diethyloxacarbocyanine iodide (DiOC_2_, BacLight bacterial membrane potential kit, and Molecular Probes/Invitrogen), a membrane potential sensitive dye. For this, the cells were stained with 2.5 µM of dye for 20 minutes at 37°C in the dark. Thereafter, the cells were washed with PBS to remove the extra stain, and flow cytometer data were acquired for 10,000 cells using an FL1 laser of Accuri C_6_ flow cytometer.

### ATP measurement

Relative ATP estimation was done using ATP Bioluminescence Assay Kit CLS II (Roche) following the manufacturer’s recommended protocol. Briefly, the cells were grown as ROS measurement protocol. Cells were harvested at 4,000 rpm for 10 minutes and washed with chilled PBS. To extract ATP, the cells were incubated in ATP extraction buffer (100-mM Tris-HCl, pH 7.75, and 4-mM EDTA, pH 8.0) at 100°C for 2 minutes, followed by separation of cell-free supernatant by centrifugation for 5 minutes at 1,000 *g*. ifty microliters each of the sample supernatant and luciferase reagents was added to wells in black 96-well microplate with gentle mixing, and the luminescence was measured using a luminometer.

The relative light units (RLUs) were recorded and normalized per milligram of protein. The normalized RLU’s of three independent experiments were plotted with SD.

### Statistical analysis

The data from three independent experiments were calculated as mean ± standard deviation. Most of fluorescent data are represented as normalized or fold change in MFI which is calculated by dividing treated sample to the untreated one. Either paired or unpaired *t*-test analysis (mentioned in the graph) was performed using GraphPad Prism version 8 software for analysis.

## SUPPLEMENTAL MATERIAL

Supplemental material spectrum.00175-23-s0001.doc Supplemental information and figures.

ASM does not own the copyrights to Supplemental Material that may be linked to, or accessed through, an article. The authors have granted ASM a non-exclusive, world-wide license to publish the Supplemental Material files. Please contact the corresponding author directly for reuse.
