# Supplementary material for: Unraveling the mechanistic insights of sophorolipid-capped gold nanoparticle-induced cell death in Vibrio cholerae
Source: Microbiol Spectr. 2023 Oct 9;11(6):e00175-23. doi: 10.1128/spectrum.00175-23 (PMC10715219; doi:10.1128/spectrum.00175-23)
Supplement: Supplemental material — Supplemental information and figures. [file spectrum.00175-23-s0001.doc]

# Supplementary Information

**Unraveling the mechanistic insights of sophorolipid-capped gold-nanoparticle induced cell death in *Vibrio cholerae***

Sristy Shikha^1†^, Vineet Kumar^2†^, Ankita Jain^1^, Dipak Dutta^2^, Mani Shankar Bhattacharyya^1^*

^1^Biochemical Engineering Research and Process Development Centre (BERPDC), CSIR-Institute of Microbial Technology (IMTECH), Sector-39A, Chandigarh 160036, India.

^2^Molecular Microbiology laboratory, CSIR-Institute of Microbial Technology (IMTECH), Sector-39A, Chandigarh 160036, India.

^†^Both authors contributed equally

Email: [manisb@imtech.res.in](mailto:manisb@imtech.res.in)

Phone +911726665313; Fax: +91-172-2695215.

**Synthesis and characterization of Sophorolipid capped gold nanoparticles (AuNPs-SL)**

Synthesis of Sophorolipid capped gold nanoparticles (AuNPs-SL) was done as mentioned earlier[1]. Briefly, 40 μl of sophorolipid (100 mg/ml) was added to the 10 ml of the chloroauric solution of concentration (400μg/ml, pH 5.5±0.2) with a few drops of freshly prepared sodium borohydride solution (NaBH_4_, 100 mM). The appearance of the characteristic red color of the solution indicated the presence of AuNPs-SL. The AuNPs-SL was analyzed spectrophotometrically using a dual-beam UV visible spectrophotometer (Hitachi U-2900) by measuring the absorption spectra in the 400 –700 nm range (Supplementary Fig 1A), Size, and ζ- potential of the synthesized nanoparticles were measured by using Dynamic Light Scattering (DLS) (Malvern Zetasizer) (Supplementary Fig 1B), Transmission Electron Microscopic (TEM) (JEOL 2100) was done to characterize the morphology and topology of the nanoparticles (Supplementary Fig 1C).

**Effect of DTT on AuNPs-SL activity and integrity**

**ROS and membrane potential measurement**

ROS was measured in the presence and absence of (AuNPs-SL-25 µg/ml) supplemented with DTT (3 mM) using H2DCFDA (10 µM). AuNPs-SL-25 treated cells were incubated for 3 hours in the presence and absence of DTT. For the membrane potential measurement, the cells were harvested and stained with DiOC2 for 30 minutes in dark and washed twice with PBS. The MFI acquired by the FL1 laser of the flow cytometer has been plotted. To check the effect of DTT on the growth of cells spot assay was performed with varying concentrations of AuNPs-SL ( 0 µg/ml, 25 µg/ml, 50 µg/ml, and 100 µg/ml) independently and with supplemented DTT (3 mM). For integrity assay UV spectra of AuNPs-SL were measured in the presence and absence of DTT at AuNPs-SL 75 µg/ml and 100 µg/ml.

**Effect of Iron supplementation using Mohr’s salt**

Under AuNPs-SL stress Log phase cells were diluted 1000 fold and different combinations were prepared in LB broth (100 µl) and different preparation was mixed to 100 µl diluted culture and added to 100 well honeycomb plates. The growth assay was performed in Bioscreener at 37°C for 16 hours and the OD600 was measured at every 1 hour of incubation. LB agar plate with different concentrations of AuNPs-SL (25 µg/ml), Mohr’s salt (1.5 mM), and a combination of the two was prepared. *V. cholerae* cells at log phase were diluted to a ratio of 1:10 ^2^, 1: 10 ^4^, 1:10 ^6^, and 10 µl of each was spotted on agar plates followed by overnight incubation at 37°C. The representative images of plates are given in figure 4C and Supplementary figure 4C.

**Measurement of different metal ion concentration upon AuNPs-SL stress**

To evaluate the change in the intracellular concentration of different metal ions, log phase cells were grown in the presence of AuNPs-SL (10 µg/ml) for 2-3 hours, harvested, and washed twice with 1X PBS (pH 7.2). Metal ion concentration within the cell pellets (20 mg) was determined by using an ICP-MS machine at (Punjab Biotechnology Incubator, Mohali, India). The cell pellet was digested with 5 ml of concentrated nitric acid and 0.5 ml hydrogen peroxide (30%). The final volume of the Digested sample was adjusted to 50 ml with deionized water and the sample was analyzed by ICP-MS. The amount of ion present was reported in mg/kg of cell pellet.

**Growth of culture in AKI condition**

AKI condition was maintained as described earlier (SOUMEN CHAKRABORTY, et al 2000). The primary culture of *Vibrio cholerae* was prepared in LB broth from an agar plate. Then, secondary inoculation was performed in AKI media AKI (containing [per liter] Bactopeptone, 15 g; NaCl, 5 g; yeast extract, 5 g; sodium bicarbonate, 3 g; pH 7.5 and incubated as required for AKI condition. After completion of incubation time, OD_600_ was measured and diluted 1000 times. 100 µl of this log phase culture was mixed with the different concentration (serially two-fold diluted 6.25- 200 μg/ml, of the AuNPs-SL & SL in 96 well plate for the antimicrobial activity. Concentrations of AuNPs-SL & SL have been mentioned in terms of SL amount. Negative (without any treatment) and positive controls (ampicillin serially two-fold diluted 3.125- 100 μg/ml) were taken. The plate was incubated at 37°C for 16 hours in the shaker at 37°C. After completion of incubation time, colorimetric XTT reduction assay was performed to know the viability of microbial cell as mentioned earlier. Micro-well plate was added with 30 µg/ml of XTT (0.5 mg/ml, Sigma-Aldrich) and incubated at 37°C in the dark for 2 hours. The reduction in formazan, a colored product formation indicated to cellular metabolic activity that was measured at 490 nm with a microplate reader (Biotek Spectrophotometer, Power wave XS2). All the experiments were performed in triplicates and average values are reported with standard deviation.

**List of Figures**

**
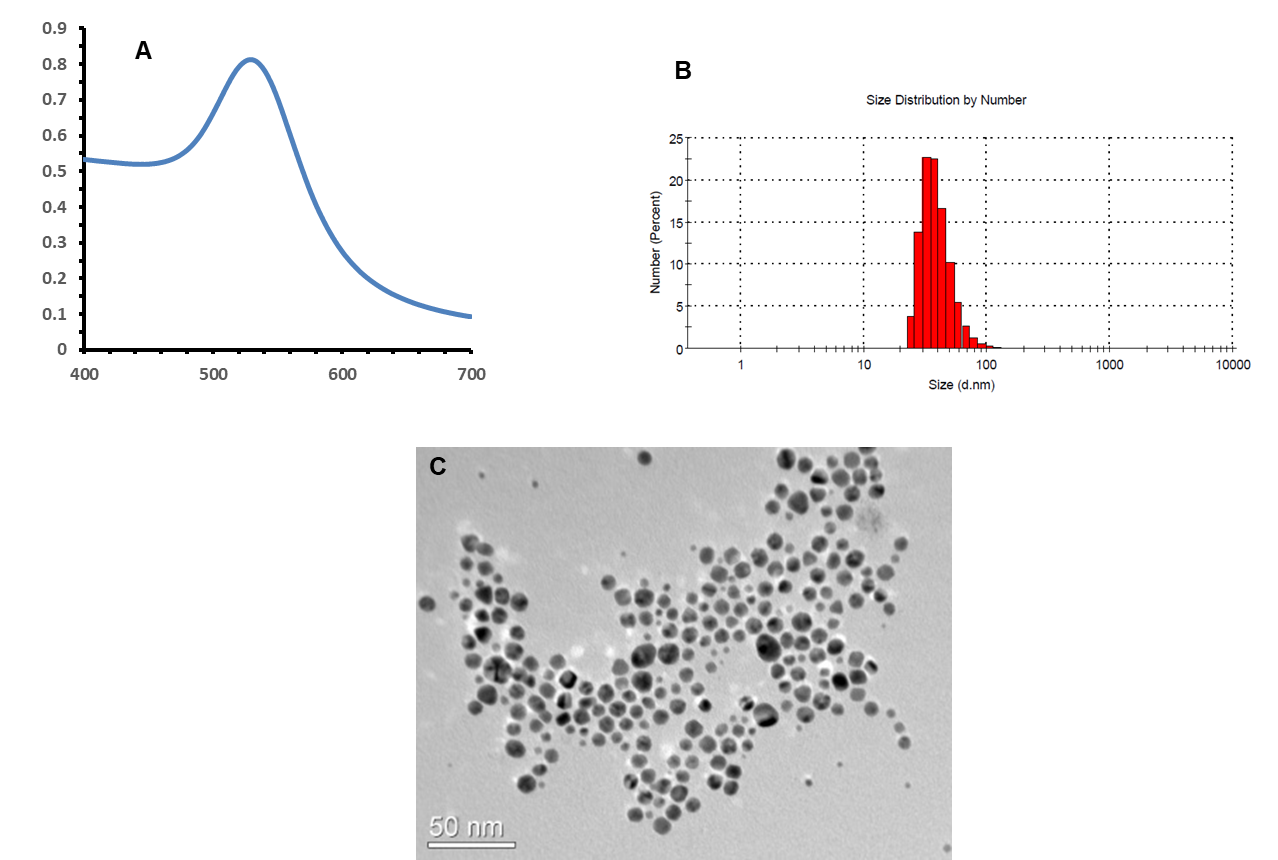
**

**Supplementary Figure 1, Characterization of AuNPs-SL nanoparticles: (A)** UV-Visible spectra (**B)** DLS result of a representative sample of AuNPs-SL **(C)** Transmission electron micrograph of the same sample.


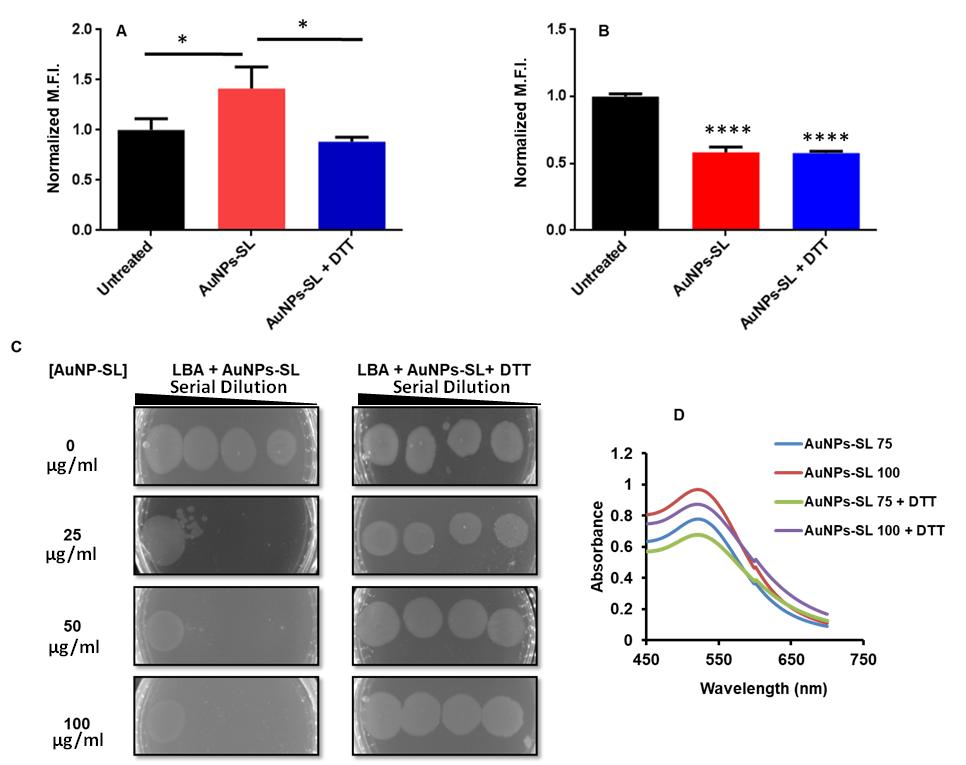


**Supplementary Figure 2**: **DTT rescues the AuNPs-SL mediated killing in *V. cholerae***. Flow cytometry analysis of ROS measurement (**A**) and membrane potential (DiOC_2_) (**B**) measurement in the presence of AuNPs-SL and DTT) (n=3± SD). P-value 0.04 for AuNPs-SL and 0.013 for AuNPs-SL in the presence of DTT **(C)** Spot assay at a varying concentration of AuNPs-SL (0 µg/ml, 25 µg/ml, 50 µg/ml, and 100 µg/ml), (**D)** UV spectra measurement of AuNPs-SL in the presence and absence of DTT indicating an insignificant change in spectral shift thus, suggesting no agglomeration of nanoparticles.

RT-qPCR


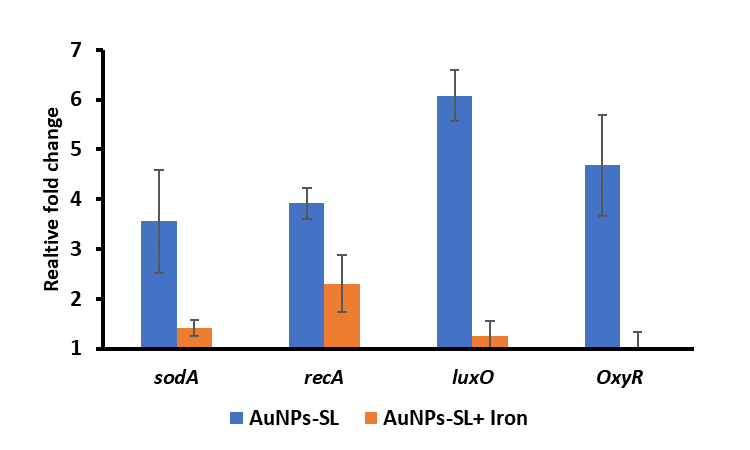


**Supplementary Figure 3**: **: RT-qPCR in AuNPs-SL nanoparticles treatment**

Fold change of selected genes in the presence of AuNPs-SL-25 µg/ml as compared to untreated samples and in combination with Mohr’s salt (ammonium iron (II) sulphate). The data of biological duplicates has been plotted with ± SD.


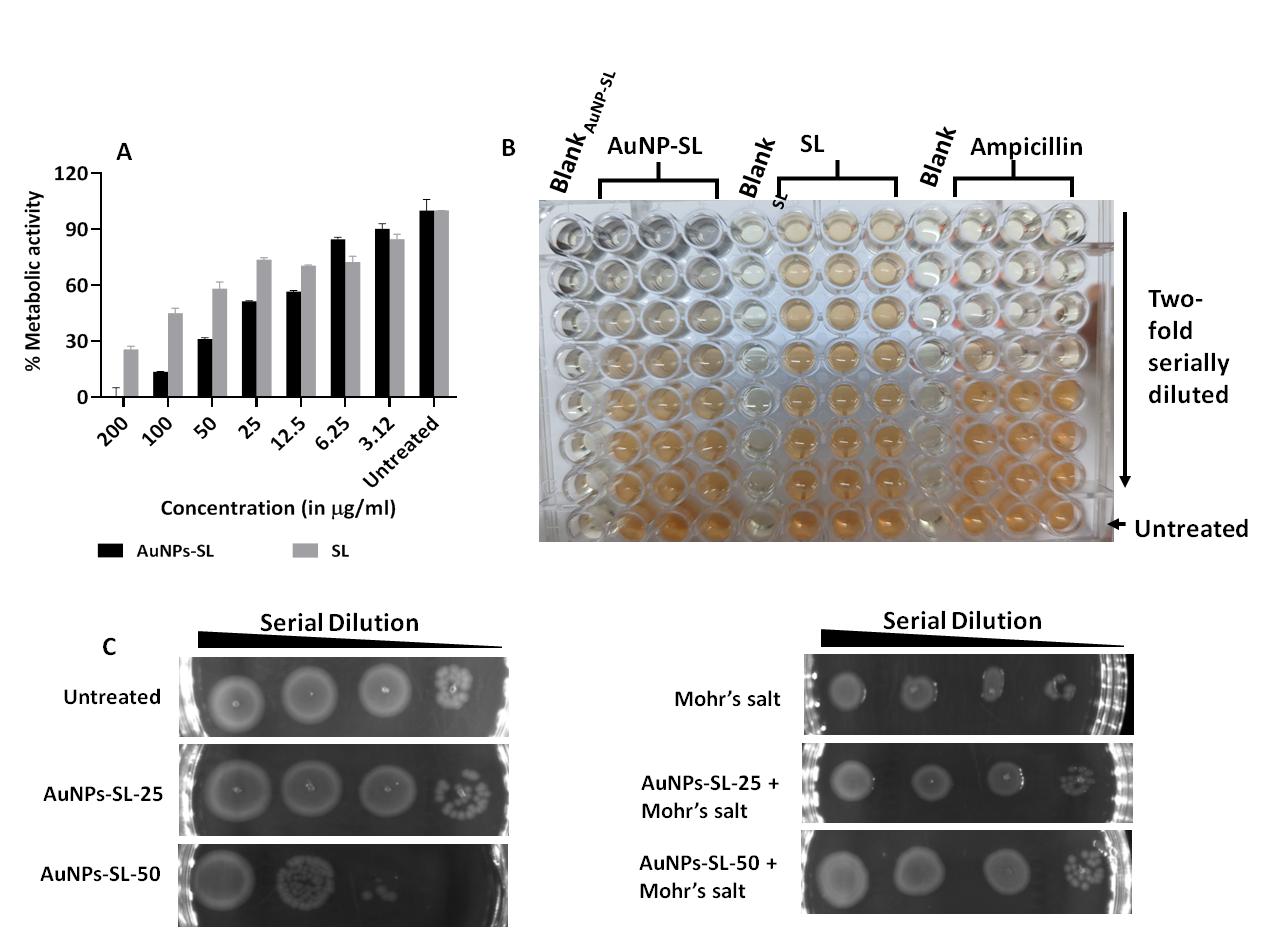


**Supplementary Figure 4**: **Antimicrobial effect of AuNPs-SL in AKI condition of *V. cholerae***. XTT Assay for metabolic survival of cells in presence AuNP-SL & SL (**A**) Microtitre plate demonstrates MIC value using XTT assay with proper blank to avoid interference imposed by AuNP-SL & SL (**B**) Spot assay at a varying concentration of AuNPs-SL (0 µg/ml, 25 µg/ml and 50 µg/ml) and iron (Mohr’s Salt) supplementation **(C)**.

**Reference**

1. Shikha S, Chaudhuri SR, Bhattacharyya MS. Facile One Pot Greener Synthesis of Sophorolipid Capped Gold Nanoparticles and its Antimicrobial Activity having Special Efficacy Against Gram Negative Vibrio cholerae. Sci Rep. 2020;10.
